# Affecting Microstructure and Properties of Additively Manufactured AISI 316L Steel by Rotary Swaging

**DOI:** 10.3390/ma15186291

**Published:** 2022-09-09

**Authors:** Lenka Kunčická, Radim Kocich, Marek Benč, Jiří Dvořák

**Affiliations:** 1 Institute of Physics of Materials, Czech Academy of Sciences, Žižkova 22, 61600 Brno, Czech Republic; 2 Faculty of Materials Science and Technology, VŠB–Technical University of Ostrava, 17. listopadu 2172/15, 70800 Ostrava, Czech Republic

**Keywords:** 316L stainless steel, selective laser melting, rotary swaging, microstructure, mechanical properties

## Abstract

The presented work focused on the development of the microstructural and mechanical properties of a AISI 316L stainless steel workpiece prepared through additive manufacturing and subsequently processed by hot rotary swaging. In order to characterize the effects of swaging on the structural development, samples were taken for electron microscopy scanning and microhardness measurements were taken after each swaging reduction. The as-built and final swaged pieces were also subjected to tensile testing at room temperature and at 900 °C. The structural analyses showed that the hot swaging introduced a substructural formation; low angle grain boundaries prevailed over high angle ones after each pass. The swaging also imparted an almost complete elimination of the porosity and significant grain size; the average grain area decreased from the original value of 365.5 µm^2^ to 4.4 µm^2^ after the final swaging pass. The changes in the texture between the passes were negligible, however, the grain refinement went hand in hand with the microhardness increase (up to almost 300 HV1). The results of the tensile testing confirmed that the mechanical properties of the swaged pieces which improved dramatically and remained favorable up to high temperatures.

## 1. Introduction

Additive manufacturing (AM), also known as 3D printing, involves numerous alternative production methods suitable to the manufacture of components with challenging shapes, based on a computer-aided design (CAD). Selective laser melting (SLM) is one of the powder bed fusion (PBF) AM processes whereby the metal powder used is melted with a powerful laser and subsequently solidified layer by layer [1,2,3,4]. The PBF process is favorably used for the production of components made from various metallic materials, e.g., Al-based [5], Ti-based [6], and Cu-based alloys [7], and numerous types of steel [2,4]. The different types of steel prepared via AM are materials with versatile applications that can be used to produce highly-resistant final components (e.g., machining tools [8]), as well as to fabricate composite and bimetallic materials [9]. For example, Kim et al. [10] used the PBF process of directed energy deposition (DED) to prepare a functionally graded material combining 316L stainless steel with Inconel 718.

The AM has many advantages, among which the greatest ones are rapid production, as well as the ability to create components of complex geometries and functional prints. However, it also features disadvantages, such as the size limitations of 3D-printed components (primarily determined by the printing chamber dimensions), their surface roughness, and the presence of internal printing defects and voids [11]. Given by the number of influencing factors affecting a 3D-printed workpiece (e.g., conditions of melting, heating, and solidification), the microstructural heterogeneity and the anisotropy of properties occur virtually within every manufactured component. Moreover, the repeated melting/solidification process typically introduces the presence of (high) residual stress, which can cause undesirable deformations in the shape and dimensions of the component, especially when non-homogeneously distributed [12]. Therefore, the post-processing can favorably be included to homogenize the as-built structures [13]. The post-processing usually consists of operations involving heat, such as a heat treatment or hot isostatic pressing. Despite the fact that the heat treatment is able to (favorably) affect structures, as well as to more or less homogenize the distribution of the residual stress, it can have a negative effect on the grain size [14]. Annealing can also introduce an undesirable precipitation and, moreover, it cannot fully eliminate the porosity and the presence of clusters of pores [15]. Therefore, additional post-processing is required to homogenize the as-built structures.

One of the possibilities to reduce (eliminate) the occurrence of printing defects and, simultaneously, to improve the performance of the components produced from SLM-manufactured materials is to combine the AM with technologies of plastic deformation. Several researchers used severe plastic deformation (SPD) methods to improve the structures and performance of as-built materials (e.g., friction stir processing/welding (FSP/FSW) [16,17], high pressure torsion (HPT) [18,19], and equal channel angular pressing (ECAP), and related methods [20,21,22,23]), especially with regards to Al-based SLM-manufactured alloys. Hosseinzadeh et al. [24] reported that when the porosity was eliminated, the grain size significantly decreased, and both ductility and the yield strength increased by more than 50% for an Al-based alloy processed via four ECAP passes. However, the applicability of the SPD methods on an industrial scale is limited given the maximum possible dimensions of usable samples. Nevertheless, other industrially applicable forming technologies can advantageously be used to impart a favorable grain refinement and structural homogenization. For example, hot forging is suitable to reduce the porosity of additively produced preforms, as well as to refine their microstructures. The occurring dynamic of recrystallization and the pressure bonding of cavities result in a significant improvement in the ductility and increase in the ultimate tensile strength. Pruncu et al. [25] documented that hot forging eliminated defects and imparted an enhanced mechanical strength, ductility, and improved isotropy within a 3D-printed steel workpiece, whereas Bambach et al. [26,27] reported that the compressive stress present during the hot forging was favorable to close internal voids and enhance microstructures of the Ti6Al4V alloy prepared via SLM.

Among the industrially applicable methods suitable to produce long bars are the radial shear rolling [28] and rotary swaging [29,30] methods. Radial shear rolling imparts the helicoidal flow to the processed materials, which advantageously enables the substantial reduction in porosity, as well as a significant grain refinement. It has also been shown to increase fatigue resistance of Ti-based alloys via the formation of dynamically polygonised dislocation substructures [31]. Rotary swaging (RS) is an intensive plastic deformation method used (in the industry) to gradually reduce cross-sections and increase the lengths of workpieces. The method is based on the repeated imposition of a severe shear strain into the processed materials in order to enhance their structures and properties. For RS, the deformation tool consists of a set of swaging dies, the acting shape of which can be designed according to the required final product shape [32]. Therefore, the method can advantageously be used to create complicated long, solid or hollow axisymmetric products.

The aim of the presented work was to characterize the effects of rotary swaging performed under hot conditions in the development of microstructural and mechanical properties of a SLM-prepared AISI 316L stainless steel workpiece. A 316L stainless steel was selected as it is a well-known and widely applicable material, e.g., for components used in gas transportation, refineries, chemical and petrochemical plants, constructions, automobiles, nuclear reactors, biomedical engineering, etc. [33,34]. In order to assess the dynamic (sub)structural development, the detailed structural observations and microhardness measurements were performed after each swaging pass. The porosity before swaging and after the final pass was evaluated via X-ray micro-computer tomography (μCT). The final swaged rods were also subjected to tensile testing at room and elevated temperatures.

## 2. Materials and Methods

The material for the experiment was prepared using the selective laser melting (SLM) method using a Renishaw AM400 3D printer (Renishaw plc., Wootton-under-Edge, UK). The original 316L stainless steel powder (by Renishaw plc., Wootton-under-Edge, UK) featured a particle size ranging from 15 to 45 µm. The powder was melted with a laser with a power of 200 W in an inert Argon atmosphere. The parameters used to build the workpiece were of a layer thickness of 50 μm, a focus size of 70 µm, and a scanning speed of 650 mm·s^−^^1^. The SLM workpiece, with the diameter of 30 mm, was built with the longitudinal axis parallel to the building substrate from left to right using the meander printing strategy [35]. Given the orientation of the workpiece in the printing chamber, its cross-section was selected to be hexagonal instead of circular in order to prevent any possible post-print shape deformations due to residual stress. Immediately after printing, the SLM workpiece was subjected to a heat treatment at 900 °C for 15 min to reduce the presence of any residual stress. The 3D-printed and subsequently heat-treated workpiece was considered as the initial *SLM sample* for the subsequent experimental study.

**Figure 1:**
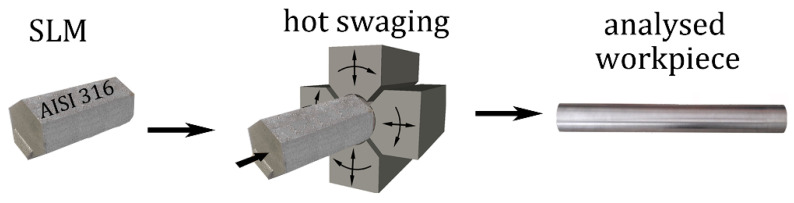
Schematic depiction of the experimental swaging process.

Then, a multi-pass deformation via rotary swaging at the temperature of 900 °C was performed (see Figure 1 for the schematic depiction of the experimental process). The swaging was carried out in four individual swaging passes from the original diameter of 30 mm to the final swaged piece diameter of 10 mm. Prior to the first pass, the edges of the workpiece were chamfered. The swaging ratios after each swaging pass were calculated via Equation (1),

(1) $$\varphi =\ln\left(\frac{S_{i}}{S_{n}}\right)$$

where *Si* and *Sn* are cross-section areas at the input and output of the swaging dies, respectively. The swaging ratios for the individually examined samples were 0.35 for *sample 1*, 0.80 for *sample 2*, 1.40 for *sample 3*, and 2.20 for *sample 4*.

The porosity within the *SLM workpiece* and *sample 4* was examined at room temperature via the X-ray micro-computer tomography (μCT) method (GE phoenix v|tome|x m 300 device with a DXR250 flat panel detector and 300 kV/500 W microfocus X-ray tube, all manufactured by Baker Hughes Digital Solutions GmbH, Hürth, Germany). The measured specimens had the dimensions of 5 × 5 × 5 mm^3^ and were taken from the axial regions of the SLM-manufactured and swaged pieces (this location was selected as the residual porosity was expected to be the highest within the material volume of the piece, especially for the swaged piece). The exposure time of the detector was 333 ms, the acceleration voltage was 200 kV, and the current of the X-ray tube was 140 μA. The tomographic reconstruction of the scanned data was performed using our own developed evaluation software, while the visualization of the internal void structures was carried out using VG Studio MAX 3 software (version 3.0 by Volume Graphics GmbH, Heidelberg, Germany).

The samples taken after each swaging pass were subjected to structural analyses using the scanning electron microscopy (SEM), in particular, the electron backscatter diffraction (EBSD) method. The observations were made using a Tescan Lyra 3 XMU FEG/SEMxFIB microscope equipped with a Symmetry EBSD detector (Tescan Orsay Holding a.s., Brno, Czech Republic). The samples were mechanically ground on SiC grinding papers and finally electropolished. The EBSD scanning was performed on samples tilted by 70°. For the detailed analyses of the grains, the scans with an area of 100 × 100 µm^2^ and a scan step of 0.1 µm were acquired, while the larger scans with an area of 200 × 300 µm^2^ and a scan step of 0.25 µm were made for the texture and grain size analyses. The EBSD analyses were evaluated via Aztec Crystal software (version 2.2, Oxford Instruments, Abingdon, UK). The texture analyses were evaluated with the maximum deviation of 15°, and the limiting value for the low angle grain boundary (LAGB) and high angle grain boundary (HAGB) was 15°.

The microhardness after each pass was measured with the load of 1000 g (HV 1) along two perpendicular diagonals on a cross-sectional sample, using a Zwick Roell machine (Zwick Roell CZ s.r.o., Brno, Czech Republic). The tensile testing of the final swaged pieces at room temperature, also at 900 °C, was performed with the strain rate of 10^−3^ s^−1^ using a Zwick/Roell KAPPA DS 50 kN testing machine (Zwick Roell CZ s.r.o., Brno, Czech Republic). For the experiment carried out at room temperature, we used an optical extensometer. During the testing, the testing machine was coupled with a digital image correlation (DIC) system that can digitally measure the longitudinal strain of the tensile specimens by means of two CCD cameras. The surface of each specimen was sprayed with a randomized pattern (painted with white background and scattered with black dots).

## 3. Results and Discussion

### 3.1. Analyses of the Grains

**Figure 2:**
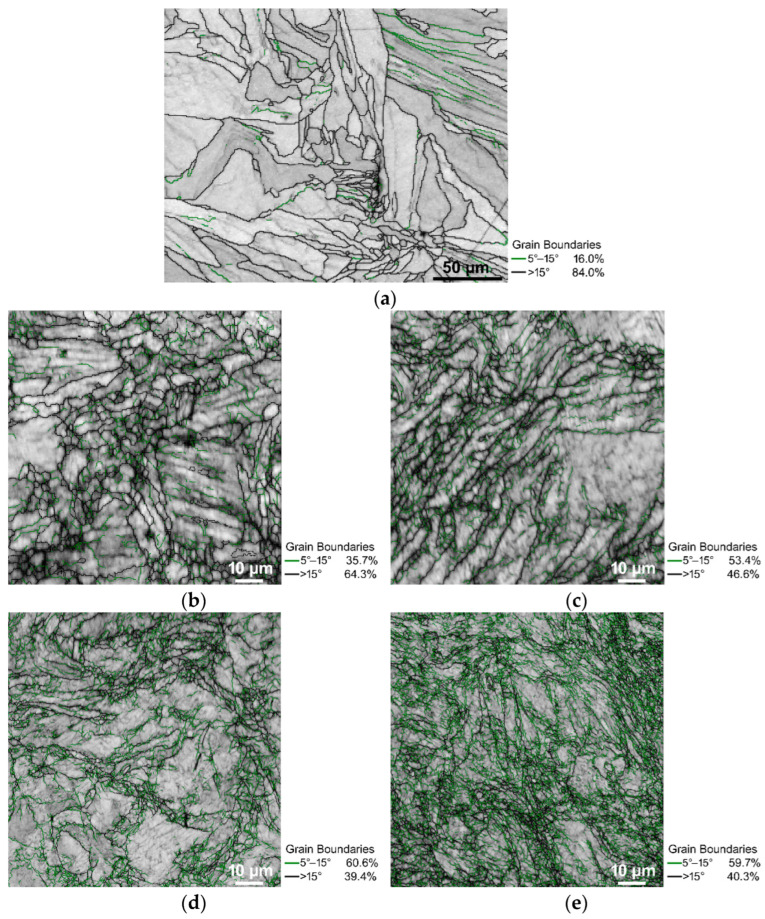
EBSD band contrast images with HAGBs depicted in black, and LAGBs depicted in green: *SLM sample* (**a**); *sample 1* (**b**); *sample 2* (**c**); *sample 3* (**d**); *sample 4* (**e**).

The microstructures of the examined samples can be seen in Figure 2a–e, the band contrast EBSD images with the HAGBs are depicted in black and the LAGBs are depicted in green. The scans were acquired using cross-sectional cuts, at a distance of 0.5 mm below the periphery of each sample as the effect of the imposed shear strain was expected to be the highest in the peripheral regions.

As can be seen, the *SLM sample* (Figure 2a) consisted mainly of fully developed grains defined with HAGBs. The fraction of the HAGBs then significantly decreased after the first swaging pass (compare 84% HAGBS for the *SLM sample* with 64.3% HAGBs for *sample 1*), and then decreased again after the second swaging pass; *sample 2* exhibited 46.6% of HAGBs. During the following two passes, the HAGB fractions slightly decreased and remained more or less comparable. In other words, the fractions of the LAGBs increased to approximately 60% for *samples 3* and *4* (Figure 2d,e).

The *SLM* material state consisted mainly of HAGBs (Figure 2a), which was expected as it was an as-printed heat treated material. Following the beginning of the swaging, the fraction of the HAGBs decreased significantly at the expense of the LAGBs, which could be attributed to the development of the substructure. The following swaging pass then caused another decrease in the HAGB fractions; this decrease was comparable with that observed after the first swaging pass (compare the HAGB fractions of 84% for the *SLM sample*, 64.3% for *sample 1*, and 46.6% for *sample 2*—Figure 2a–c). Following the second swaging pass, the structure was saturated and the development of the substructure and the dynamic restoration were equalized by the effect of which the fractions of the HAGBs did not significantly decrease during the continuing swaging (*samples 3* and *4* exhibited comparable HAGBs fractions—Figure 2d,e).

**Figure 3:**
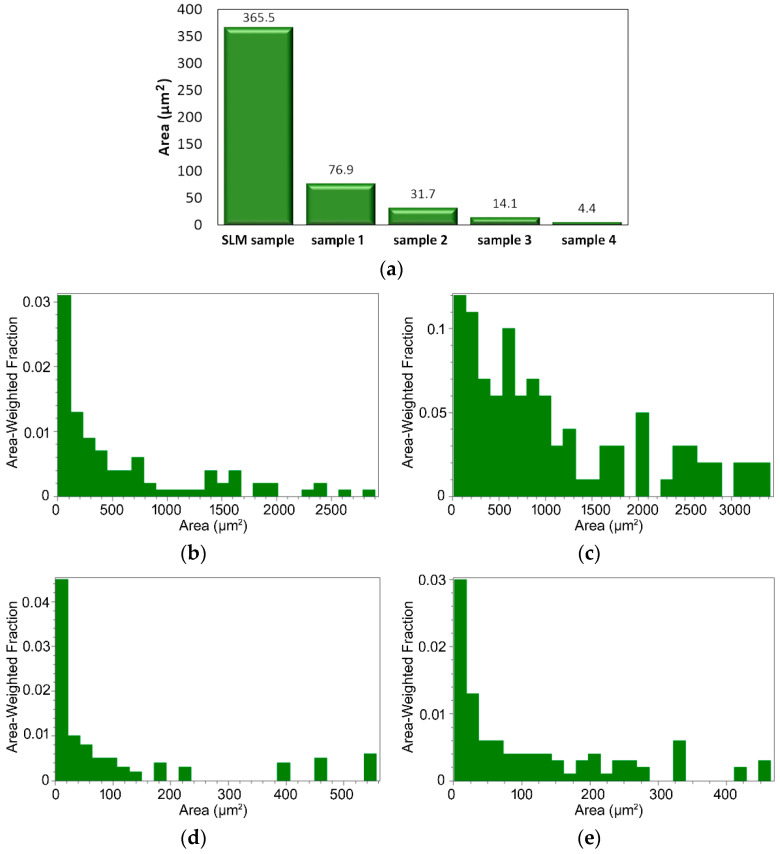
Average grain areas for the examined samples (**a**). Grain area distributions for: *sample 1* peripheral region (**b**); *sample 1* axial region (**c**); *sample 4* peripheral region (**d**); *sample 4* axial region (**e**).

As is evident from Figure 2a–e, especially in the structures of the pieces swaged with a lower reduction, the ratios were mostly bimodal. For this reason, the grain size was evaluated via the average grain area parameter (in μm^2^), rather than via the average grain diameter (in μm). The results of the grain size analyses for the peripheral areas of all of the examined samples are summarized in Figure 3a showing that the swaging affected the grain size favorably. The average grain area dramatically decreased during the swaging, down to slightly less than 4 μm^2^ after the final swaging pass. In order to assess the effect of the imposed shear strain on the structural homogenization, the analyses of the peripheral regions were supplemented with analyses of the axial regions for the samples subjected to the first and last swaging passes, i.e., *sample 1* and *sample 4*. As is evident from Figure 3b and c that depict the grain size distributions in the peripheral and axial regions of *sample 1* via the area-weighted fractions, the grains in the axial region were evidently larger than in the peripheral region. A similar phenomenon was observed in *sample 4* (Figure 3d,e). The continuous substructural development contributed to the significant grain size decrease, as the grains evidently underwent an intense fragmentation. This phenomenon was supported by the mutual effect of the intensive imposed shear strain and elevated temperature [36]. Although the maximum grain areas observed within the structures were significantly higher than the average values, they continuously decreased during the swaging process—from 15,200 μm^2^ for the *SLM sample*, to 3240 μm^2^ for *sample 1*, 3005 μm^2^ for *sample 2*, 2780 μm^2^ for *sample 3*, and 580 μm^2^ for *sample 4* (the maximum values acquired from the analyses of the scans were taken in all of the examined areas).

The 3D-printed material consisted of a mixture of large and small grains, i.e., a bimodal structure. Many of the grains were elongated along a single direction (Figure 2a), which gives rise to a hypothesis that this structure developed correspondingly to the heat transfer during the 3D printing. In other words, some of the grains had favorable orientations from the viewpoint of the heat dissipation during the printing, which provided them with advantageous conditions for the (abnormal) growth [37]. However, some of the grains had aggravated conditions for their growth and needed a higher activation energy. This could have been caused by multiple factors, such as the aggravation of grain boundary migration due to obstacles, e.g., voids and printing defects, or rapidly growing adjoining grains resulting in the development of triple junctions with a very low migration ability (similar structural phenomena could have also caused the bimodal grain size distribution within the swaged pieces) [38].

### 3.2. Texture

**Figure 4:**
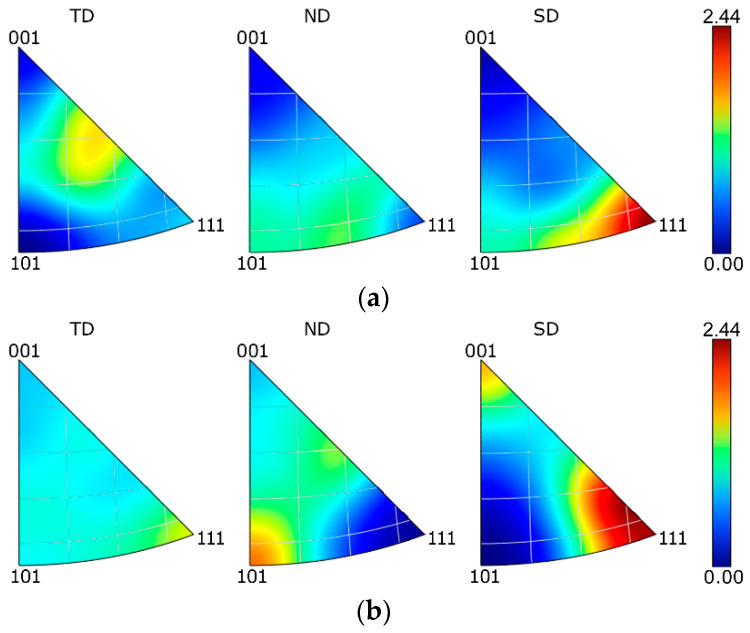

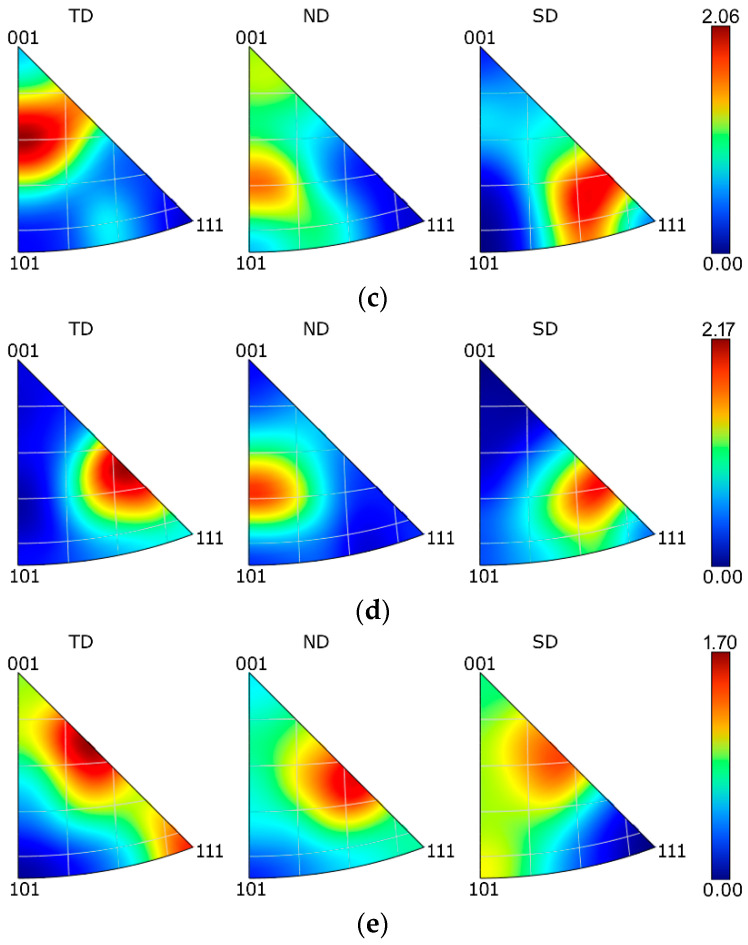
IPF texture images for: *SLM* sample (**a**); *1* sample (**b**); *2* sample (**c**); *3* sample (**d**); *4* sample (**e**).

The results of the textural analyses acquired at the peripheral regions of cross-sectional cuts through the swaged bars in the *z* axis, i.e., the longitudinal sample axis, are depicted via Inverse Pole Figures (IPFs) in Figure 4a–e. The orientations of the crystallites within the *SLM sample* were more or less random (Figure 4a). The sample featured the tendency to form the <111>||SD preferential texture (with a shift towards the <101>||SD orientation), but its maximum intensity was not very high (slightly higher than two times random). The first swaging pass did not significantly affect the grains’ orientations, as the maximum texture intensity remained more or less unchanged (Figure 4b). However, some of the crystallites exhibited the tendency to form the <001>||SD preferential orientation. Further swaging contributed to texture randomization, as the maximum texture intensity slightly decreased after the second swaging pass (Figure 4c). Furthermore, the tendency to form the <111>||SD texture fiber was reduced and the preferential orientations of the grains shifted by approx. 20° towards the <001>||SD orientation, and 30° towards the <101>||SD orientation for *sample 2*. Continuing the swaging further contributed to the texture randomization. Both *samples 3* and *4* (Figure 4d,e) exhibited a shifting of the <111>||SD preferential texture orientation towards <001>||SD; the highest texture intensity after the final swaging pass was observed approx. 45° between these preferential orientations. The hot swaging process thus contributed to the shifting of the <111>||SD orientation towards the <001>||SD orientation and the decrease in the maximum texture intensity when compared with the *SLM sample*. The last swaging pass did not introduce any significant texture changes. The IPF images for the TD and SD directions were comparable, although a slight change was observed for the ND direction (Figure 4d,e). The textural analyses thus did not reveal any evident formation of any prevailing ideal texture orientation during the swaging.

### 3.3. Porosity

**Figure 5:**
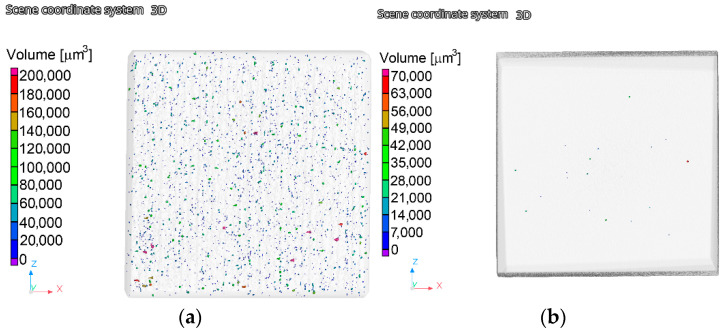
Results of the analyses of the porosity: *SLM* sample (**a**); *4* sample (**b**).

The analyses of the porosity were performed after the SLM manufacturing, as well as after the finalization of the swaging. A visualization of the results was performed along an axial cutting plane superimposed through a cubical scanned sample for each examined material state. As is evident from Figure 5a,b that depict the results for the *SLM sample* and sample 4, respectively, the effect of the rotary swaging on the elimination of the porosity was highly positive.

In other words, the amount of pores within the swaged sample was negligible when compared to the *SLM* sample. Nevertheless, not only did the amount of pores decrease dramatically after the swaging, but the two examined samples also differed from the viewpoint of the volume of the pores. Whereas the *SLM sample* contained relatively huge pores with volumes up to 200,000 μm^3^, the swaging resulted in a significant decrease in the pore size as the maximum detected pore volume within the *sample 4* was ~70,000 μm^3^. Moreover, the presence of such voluminous pores within the swaged sample was scarce. The overall decrease in porosity—compare the *SLM sample* with *sample 4* —was by ~98%; the pore size had decreased by ~65% after the swaging.

### 3.4. Mechanical Properties

**Figure 6:**
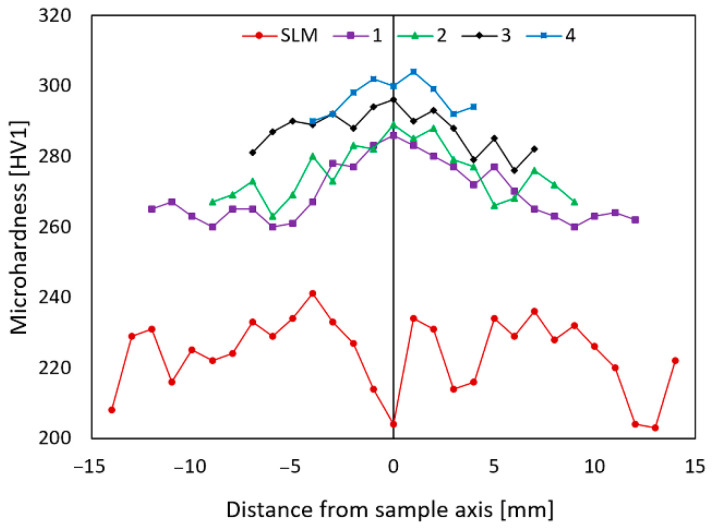
Distributions of the HV1 microhardness values across the cross-sections of the examined samples.

The results of the microhardness measurements performed for each examined material state are summarized in Figure 6 that graphically depicts the distribution of the HV1 values on the diagonals across the cross-sections of the individual samples. The *SLM* sample evidently exhibited the lowest microhardness out of all of the examined samples (average value of 224 HV1), the values acquired for this material state also featured the highest observed standard deviation of 10.2 with local drops in the locations of the printing defects (pores). The continued swaging then contributed to a gradual microhardness increase up to a final average value of 295 HV1 that was acquired for the final swaged rod, i.e., *sample 4*. The average HV1 values for *sample 1* and *sample 2* were comparable (270 HV1 and 272 HV1, respectively), and for *sample 3*, it reached 286 HV1. The standard deviation decreased to 8.1 for *sample 1* and decreased to 7.6 for *sample 2*, 5.5 for *sample 3*, and to 4.6 for *sample 4*.

The fact that the *SLM sample* featured the lowest acquired microhardness can be attributed to several factors. As this sample was the “raw” as-printed material, it contained numerous printing defects and voids (Figure 5a), which also corresponds to the relatively high HV1 standard deviation value calculated for this sample. Furthermore, the material was heat treated before swaging, which is known to generally reduce the lattice defects and to improve the ductility at the expense of hardness, i.e., strength. Considering all the examined samples, the highest microhardness increase was detected to have occurred during the first swaging pass. This phenomenon could be attributed to the positive effect of swaging on the closing of printing defects and on the development of the substructure, which was initiated with a high intensity due to the high processing temperature and significant imposed shear strain. Further development of the substructure and gradual grains’ fragmentation then contributed to the microhardness increase during the swaging up to the final value of ~ 300 HV1 acquired for *sample 4*.

**Figure 7:**
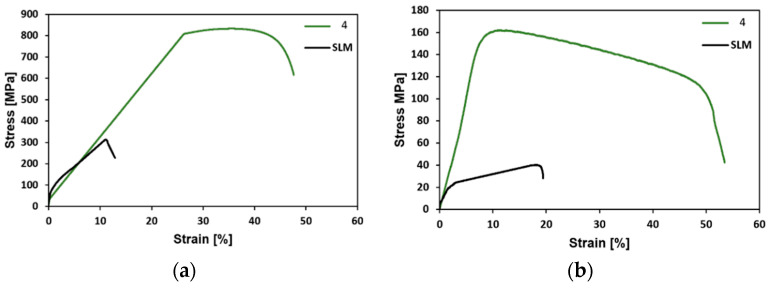
Tensile test stress-strain curves for the *SLM* workpiece and the *4* swaged piece acquired at: room temperature (**a**); 900 °C (**b**).

Last but not least, the final swaged rods were subjected to tensile testing at room temperature and at 900 °C. For comparison, the original *SLM* material was examined as well. The stress-strain curves acquired at room temperature are depicted in Figure 7a, whereas the results of tensile testing at 900 °C are shown in Figure 7b. With regards to the plasticity, the swaging evidently introduced a significant increase in this parameter. The elongation for failure was lower than 20% for the *SLM sample* even when tested at 900 °C, whereas the ductility of the final swaged rod was already at almost 50% at room temperature, and exceeded 50% when tested at 900 °C. The swaged rod also exhibited a substantial increase in strength; the room temperature ultimate tensile strength (UTS) of the *SLM sample* was 314 MPa, whereas the final swaged piece (*sample 4*) exhibited the room temperature UTS of more than 800 MPa. At the elevated temperature of 900 °C, the UTS of *sample 4* still reached up to more than 160 MPa, while the UTS of the *SLM sample* was as low as 40 MPa.

**Figure 8:**
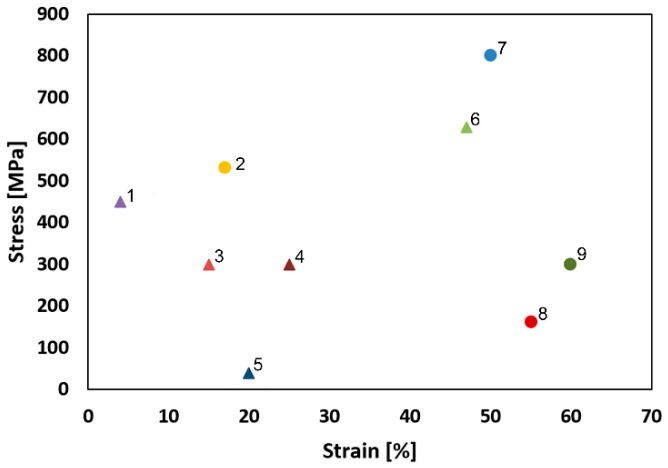
Comparison of the aquired results of the mechanical properties (UTS values) with UTS values of the 3D-printed and deformation-processed AISI 316L steel presented in the available literature. Legend to the individual points: 1—high laser power sample by Montero-Sistiaga et al. [39]; 2—Pruncu et al. [25]; 3—*SLM sample* at room temperature; 4—Dryepondt et al. [11]; 5—*SLM sample* at 900 °C; 6—low laser power sample by Montero-Sistiaga et al. [39]; 7—*sample 4* at room temperature; 8—*sample 4* at 900 °C; 9—Byun et al. [40].

Compared with the *SLM sample*, *sample 4* exhibited a substantial increase in strength (UTS), as well as in plasticity. The rapidly increasing mechanical properties, particularly strength, can be attributed to several factors. One of them is the observed substructural formation and corresponding increase in the dislocation density. Another factor which must be stressed upon is the development of other structural phenomena (e.g., deformation bands) that are very effective for strengthening. On the other hand, the favorable ductility of the swaged piece was imparted primarily by the extreme reduction of the porosity when compared with the *SLM sample* (Figure 5a,b). Furthermore, the 3D-printed materials feature a non-homogenous stress state even when a post-process heat treatment is applied. Usually, the tensile residual stress prevails in the (sub)surface regions of a 3D-printed part, whereas the compressive stress is localized in the remaining material volume. The presence of the residual stress, together with the observed porosity, were most probably the reasons for the relatively low UTS value acquired for the *SLM sample*—when compared to others (e.g., Pruncu et al. [25] acquired the UTS of ~450 MPa for a SLM-manufactured AISI 316L workpiece built with its axis parallel to the building substrate). It is generally known that the plasticity decreases dramatically with the increasing porosity. The hot rotary swaging, particularly given by the applied intensive shear strain, was evidently able to reduce the level of porosity, as well as favorably affect the original stress state of the 3D-printed workpiece, which resulted in the final enhancement of the overall performance of the swaged AISI 316L steel rod. The comparison with other plastic deformation methods, which can be used to post process 3D-printed AISI 316L workpieces, shows that the hot rotary swaging imparts a mechanical behavior superior to that acquired e.g., after hot forging, the acquired UTS was ~ 530 MPa and the elongation to failure was approx. 17% [25]. The depiction of the comparison of the UTS values acquired within the presented study with the results presented by others can be seen in Figure 8.

## 4. Conclusions

The study investigated the effects of hot rotary swaging on structural and mechanical properties of an AISI 316L stainless steel workpiece manufactured by selective laser melting. The swaging led to an almost complete elimination of the porosity (decrease by ~98%), which went hand in hand with the structural refinement and development of the substructure. The acquired structural modifications resulted in an increase in the microhardness up to almost 300 HV1, as well as enhanced tensile properties at both room temperature and 900 °C. The room temperature ultimate tensile strength (UTS) increased almost three times after the swaging and exceeded 800 MPa. At 900 °C, the UTS of the swaged piece was more than four times higher when compared with the as-printed workpiece. Suffice to say, the implementation of the hot rotary swaging substantially enhanced the overall performance of the as-printed AISI 316L workpiece.

## Data Availability

The original data supporting the research is not publicly available but the data that is not confidential is available upon request from the corresponding author.
